# The therapeutic effect of switching from tacrolimus to low-dose cyclosporine A in renal transplant recipients with BK virus nephropathy

**DOI:** 10.1042/BSR20182058

**Published:** 2019-02-22

**Authors:** Xu-Tao Chen, Jun Li, Rong-Hai Deng, Shi-Cong Yang, Yan-Yang Chen, Pei-Song Chen, Ze-Yuan Wang, Yang Huang, Chang-Xi Wang, Gang Huang

**Affiliations:** 1 Organ Transplant Center, The First Affiliated Hospital of Sun Yat-sen University, 58 Zhongshan Road 2, Guangzhou 510080, Guangdong Province, China; 2 Department of Pathology, The First Affiliated Hospital of Sun Yat-sen University, 58 Zhongshan Road 2, Guangzhou 510080, Guangdong Province, China; 3 Department of Clinical Laboratory, The First Affiliated Hospital of Sun Yat-sen University, 58 Zhongshan Road 2, Guangzhou 510080, Guangdong Province, China; 4 Zhongshan School of Medicine, Sun Yat-Sen University, 74 Zhongshan Road 2, Guangzhou 510080, Guangdong Province, China

**Keywords:** BK virus, BK virus nephropathy, cyclosporine A, conversion of immunosuppression, tacrolimus

## Abstract

**Background:** There is no effective therapy for BK virus (BKV) nephropathy (BKVN). Cyclosporine A (CsA) has a lower immunosuppressive effect than tacrolimus. *In vitro* studies have shown that CsA inhibits BKV replication. The present study aimed to evaluate the effectiveness of switching from tacrolimus to low-dose CsA in renal transplant recipients with BKVN. **Methods:** Twenty-four patients diagnosed with BKVN between January 2015 and December 2016 were included. Tacrolimus was switched to low-dose CsA, and patients were followed for 24 months. Primary end points were BKV clearance in blood and graft. Secondary end points were urine specific gravity, serum creatinine, and graft loss. **Results:** The viremia in all patients cleared at a mean of 2.7 ± 2.0 months after switching to CsA. Urine specific gravity at 3 months after switching to CsA increased significantly compared with that at diagnosis (*P*=0.002). The timing and trend of urine specific gravity increase was consistent with the timing and trend of blood and urine viral load decrease. Repeated biopsies at a median of 11.2 months (range: 9.1–12.5 months) after switching to CsA showed that 8 patients (42.1%) were negative for BKV, and 11 patients (58.9%) had a decrease in BKV load (*P*<0.001). There was no statistical difference in the serum creatinine level between the time of diagnosis and 24 months of CsA therapy (*P*=0.963). The graft survival rate was 100%. Only two patients (8.3%) suffered from acute rejection. **Conclusion:** Switching from tacrolimus to low-dose CsA may be an effective therapy for BKVN.

## Introduction

BK virus (BKV) nephropathy (BKVN) is one of the main complications affecting renal transplant function and prognosis [1–4]. BKVN occurs in 1–10% of renal transplant recipients [5] and 15–50% of patients with BKVN will develop to graft loss [5]. BKVN occurs at a higher rate during the period of tacrolimus administration than during the period of administration of cyclosporine A (CsA) [6].

BKVN is difficult to treat because there are no effective antiviral agents [3,7–9]. Published guidelines advocate regular monitoring and timely reduction in immunosuppression as the most optimal practices for BKVN [5]. Different protocols have been reported, including reducing the dose of calcineurin inhibitors, mycophenolic acid (MPA), or azathioprine, switching from tacrolimus to CsA or sirolimus, switching from MPA to azathioprine, or complete discontinuation of the antimetabolite [10]. Prospective clinical trials to validate different strategies are lacking, and published reports are unsatisfactory [3].

A new approach of conversion from tacrolimus into low-dosage CsA appears promising for several reasons. First, the immunosuppressive strength of CsA is weaker than that of tacrolimus [11]. Second, the CsA regimen has a significantly lower incidence of BK viremia and BKVN compared with the tacrolimus regimen [12–16]. Finally, *in vitro* studies indicate that CsA inhibits BKV replication by binding to cyclophilins [17,18], whereas tacrolimus activates BKV replication via FK-binding protein 12 in primary human tubular epithelial cells [19]. At present, there is only one single-center retrospective study suggesting that addition of intravenous immunoglobulin after switching from tacrolimus to CsA is helpful for clearing BK viremia [20].

Thus, the purpose of the prospective clinical study was to evaluate the efficacy and safety of conversion from tacrolimus into low-dose CsA in renal transplant recipients with BKVN.

## Materials and methods

### Study design

This was a single-center prospective clinical study with a follow-up of 24 months. The study was performed in accordance with the Declaration of Helsinki and approved by the institutional review board. The present study was registered in the Chinese Clinical Trial Registration database (No. ChiCTR-IOR-17010993).

Patients who received a renal transplant and were diagnosed with biopsy-proved BKVN between January 2015 and December 2016 were selected as candidates. Patients receiving triple immunosuppressive drugs (tacrolimus + MPA + steroid) were included. The exclusion criteria were: (i) serum creatinine > 300 µmol/l when diagnosed with BKVN; (ii) received large-dose methylprednisolone treatment within 1 month before diagnosis of BKVN; (iii) receiving antiviral drug treatment; (iv) receiving other immunosuppressant drugs. Written informed consent was obtained from all the patients.

### Clinical follow-up and data collection

All patients were followed for 24 months. The follow-up points included before switching to CsA, and then 2 weeks, and 1, 3, 6, 9, 12, 18, and 24 months after switching to CsA. Clinical indicators were recorded at each follow-up time-point. The estimated glomerular filtration rate (eGFR) was calculated with MDRD formula [21]. Repeated renal biopsies were performed 6–12 months after switching to CsA, or when the serum creatinine had increased by more than 30%.

### Urine cytological studies and virological studies

Urine cytology smears were stained by the Papanicolaou method, and evaluated for the presence of cells with intranuclear viral inclusions (decoy cells), which were counted as number per ten high-power fields [HPF] [22–24]. Quantitation of the urine and plasma BK viral load was performed by quantitative PCR (Q-PCR) and reported as copies/ml [22,23].

### Diagnosis of BKVN

BKVN was defined by positive immunohistochemical nuclear staining with anti-SV40 large T antigen monoclonal antibody, as previously described [23]. The histological features of BKVN were classified using the American Society of Transplantation (AST) schema, and assigned to BKVN categories A, B, and C based on the guidelines published by Hirsch and Randhawa [25]. Histological viral load was assessed semi-quantitatively as the percentage of tubules positive for polyomavirus using a four-tier system (<10, 10–25, 25–50, and >50%) [26]. Histologic lesions, T-cell-mediated rejection (TCMR), and antibody-mediated rejection (ABMR) were defined using the Banff 2013 schema of renal allograft pathology [27].

### Treatment of BKVN

All patients in the present study were switched to low-dose cyclosporine immediately after the diagnosis of BKVN, and no other immunosuppressant adjustment or antiviral therapy was performed. The initial dose of CsA was 3 mg/kg/day. The trough concentration of CsA was measured every 3 days, and the dose was adjusted if needed. The target trough concentration was 75–125 ng/ml. Mycophenolate mofetil (MMF) was continued at a dosage of 500 mg twice daily, the enteric-coated mycophenolate sodium (EC-MPS) was 360 mg twice daily, and the oral steroid dosage was 5 mg/d.

### Efficacy evaluation

The primary study end points were BKV clearance in blood and renal tissues. BKV clearance in blood was defined as a BKV load of <1000 copies/ml at each test (three or more tests were performed over an interval of 3 months). BKV clearance in renal tissues was defined as negative SV40-T antigen immunohistochemical staining. The secondary end points were urine specific gravity, serum creatinine, and graft loss. The normal range of urine specific gravity was 1.005–1.015. Graft loss was defined as patient death, return to dialysis, or re-transplantation.

### Safety evaluation

The safety evaluation included rejection events, proteinuria, and CsA-related adverse events.

The CsA adverse events, including gingival hyperplasia, hair hyperplasia, hypertension, CNI nephrotoxicity and liver damage was recorded.

### Statistical analysis

All statistical analyses and calculations were performed using SPSS version 20.0 (IBM, U.S.A.). Categorical variables were presented as count and percentage. Normally distributed continuous data were presented as mean and S.D.; non-normally distributed data were presented as median (interquartile range). Normality was determined using the Kolmogorov–Smirnov test. The McNemar test was used for categorical variables. The paired-samples *t* test was used to compare normally distributed continuous variables, and the Wilcoxon signed-rank test was used for non-normally distributed continuous variables. Kaplan–Meier analysis was used for calculating the cumulative virus clearance rate after switching immunosuppression. All *P*-values were two-tailed, and <0.05 were considered significant.

## Results

### Patient baseline characteristics

**Figure 1:**
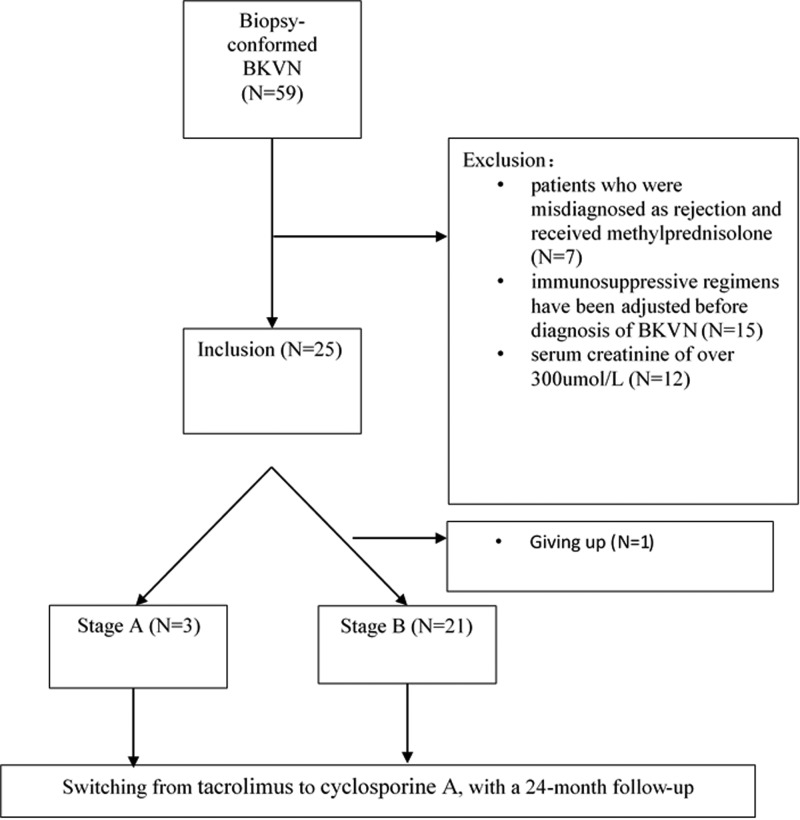
Flow diagram of patient inclusion

A total of 299 renal transplant recipients underwent 345 renal transplant biopsies between January 2015 and December 2016, and 59 patients were diagnosed with BKVN. Amongst them, 24 patients were included in the present study. The research procedure is summarized in Figure 1. Baseline data are summarized in Table 1. Amongst the 24 patients, three patients were diagnosed as stage A BKVN, and 21 patients were diagnosed as stage B BKVN. The time interval from transplantation to diagnosis of BKVN was 16.3 ± 9.4 months. Transplant data are summarized in Table 2.

**Table 1:** Baseline data of 24 patients before diagnosis of BKVN

	Total (*n*=24)
Male gender, *n* (%)	18 (75.0%)
Age at transplant (years), *n* (%)	37.5 (31.3–45.4)
Body weight (kg)	51.5 (50.0–57.0)
ESRD etiology, *n* (%)	
Glomerulonephritis	14 (58.3%)
IgA nephrology	6 (25.0%)
Hypertensive nephropathy	2 (8.3%)
Nephrotic syndrome	1 (4.2%)
Lupus nephritis	1 (4.2%)
Induction regimen, *n* (%)	
Polyclonal antibody/ATG	12 (50.0%)
Monoclonal antibody/Basiliximab	12 (50.0%)
Maintenance regimen, *n* (%)	
Tacrolimus-MPA-Steroid	24 (100%)
MPA-AUC (ng/ml)	68.0 ± 30.2
Transplant donor type, *n* (%)	
Deceased	17 (70.8%)
Living	7 (29.2%)

Abbreviations: ATG, antithymocyte globulin; MPA-AUC, MPA concentration-area under curve.

**Table 2:** Clinical data of 24 patients at the time of diagnosis of BKVN and 12 and 24 months after follow-up

	Baseline at diagnosis	Follow-up of 12 months	P*	Follow-up of 24 months	P**
Decoy cells (/HPF)	28.0 (13.8–56.8)	0 (0–0)	<0.001	0 (0–0)	<0.001
Urine BKV-DNA (copies/ml)	2.1 × 10^9^ (7.3 × 10^8^ to 5.9 × 10^9^)	5.9 × 10^6^ (1.5 × 10^5^ to 3.2 × 10^7^)	<0.001	3.7 × 10^5^ (1.0 × 10^6^ to 5.9 × 10^7^)	<0.001
Blood BKV-DNA (copies/ml)	1.4 × 10^4^ (4.5 × 10^3^ to 9.0 × 10^4^)	0 (0–0)	<0.001	0 (0–0)	<0.001
Serum creatinine (umol/l)	160.6 ± 47.3	166.5 ± 75.7	0.748	159.8 ± 88.9	0.963
eGFR (ml/min)	46.0 ± 15.5	47.4 ± 17.2	0.774	51 ± 18.3	0.252

*, *P*-value of the comparison between the time of 12 months after follow-up and diagnosis of BKVN. **, *P*-value of the comparison between the time of 24 months after follow-up and diagnosis of BKVN.

### Switching from tacrolimus to low-dose CsA

**Figure 2:**
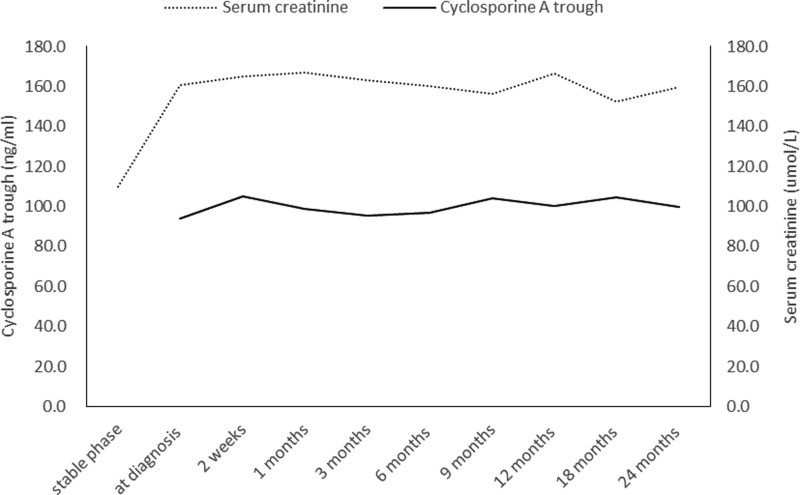
Trough concentration of CsA and serum creatinine level at each follow-up point after switching from tacrolimus to CsA were stable

All patients started with cyclosporine at a mean dose of 162.1 ± 27.2 mg/day, orally twice daily. Two weeks after switching to CsA, the mean trough concentration of CsA was 94.3 ± 33.6 ng/ml. The trough concentration remained stable during the follow-up period (Figure 2). Three patients were switched back to tacrolimus at 9, 9, and 12 months after switching to CsA. The trough concentration of tacrolimus in three patients switched back to tacrolimus set at 4–5 ng/ml, which was slightly lower than the previous trough concentration. One patient developed ABMR, and in the other two patients, the clinicians were concerned that immunosuppression was not sufficient. Before switching back to tacrolimus, blood tests for BKV had been negative for more than 3 months. None of the patients developed BK viremia or BKVN recurrence after switching back to tacrolimus.

### Efficacy evaluation

#### Switching from tacrolimus to CsA reduced the blood and urine BKV load

**Figure 3:**
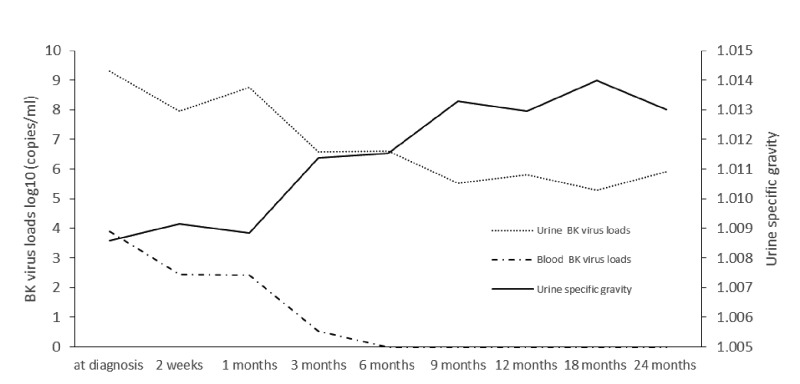
The BKV load in blood and tissues was reduced after switching from tacrolimus to CsA The timing and trend of BKV load decrease was consistent with the timing and trend of urine specific gravity increase. After log conversion, the BKV load in blood and urine, and urine specific gravity were expressed as mean.

BKV clearance is shown in Figure 3. At 3 months after switching from tacrolimus to CsA, decoy cells and blood and urine BKV load were significantly lower than those at diagnosis (all, *P*<0.001). At a mean of 2.7 ± 2.0 months after switching to CsA, all patients were negative for viremia. At a mean of 1.8 ± 1.9 months after switching, decoy cells were not present in any of the patients. Decoy cells were cleared 0.8 ± 0.8 months earlier than when viremia was cleared. Viruria was cleared in only 3 patients during follow-up.

#### Switching from tacrolimus to CsA promoted urine specific gravity recover

The mean value of morning urine specific gravity was 1.0086 ± 0.0027 at diagnosis of BKVN. In contrast, the mean morning urine specific gravity at 3 months after switching to CsA increased significantly (1.0114 ± 0.0032 compared with 1.0086 ± 0.0027, *P*=0.002). The timing and trend of urine specific gravity increase was consistent with the timing and trend of blood and urine viral load decrease (Figure 3).

#### Renal histopathological changes after switching from tacrolimus to CsA

**Figure 4:**
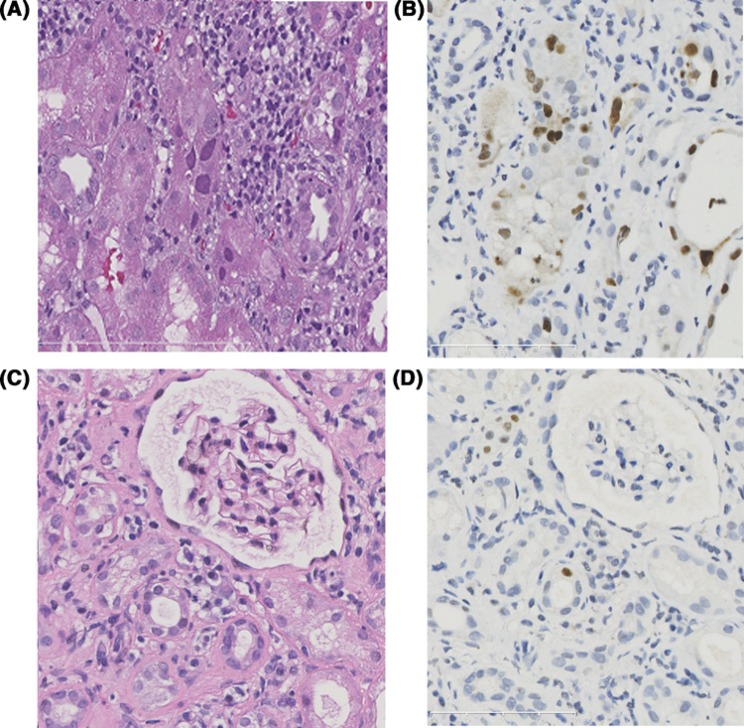
Comparison of representative pathological findings between initial biopsy and repeated biopsy BKV cytopathic changes and SV40 large T antigen staining before (**A**,**B**) and after (**C**,**D**) treatment. Initial biopsy show nuclear enlargement and nuclear inclusions within tubular epithelial cells (A). The SV40 large T antigen staining (B) shows extensive staining in the nuclei in the infected tubules. After switching to low-dose cyclosporine, the viral cytopathic changes become sparse or unrecognizable on Hematoxylin and Eosin staining. SV40 large T antigen staining (D) shows infected nuclei in isolated cells.

**Figure 5:**
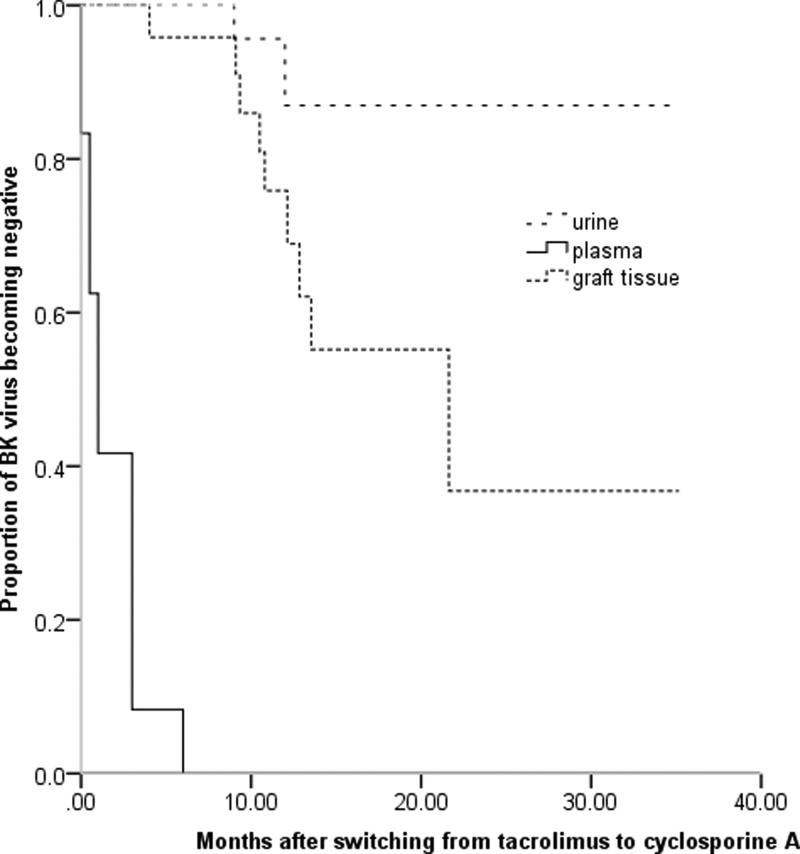
The BKV clearance in urine, blood, and tissues after switching from tacrolimus to CsA

At a median of 11.2 months (range: 9.1–12.5 months) after switching to CsA, 19 patients received 22 renal biopsies. The pathological scores of the first and last renal biopsies are shown in Table 3. The last biopsies showed that SV40-T staining was negative in 8 patients (42.1%), and was decreased in the other 11 patients (58.9%). Representative Hematoxylin and Eosin staining and immunohistochemical staining results before and after treatment are shown in Figure 4. The time of clearance of SV40-T staining was 10.7 months (range: 8.2–21.6 months). The timeline of virus clearance is shown in Figure 5. Five patients did not receive repeated renal biopsies, but their renal function was stable. Their median serum creatinine at the last follow-up was 102.0 µmol/l (range: 87.0–118.0 µmol/l).

**Table 3:** Pathological characteristics at BKVN diagnosis and last repeated biopsies

	Initial biopsy, *n*=24	Last biopsy, *n*=19	*P*
SV40-T, *n* (%)	24 (100%)	11 (57.9%)	0.002
Banff t score	1.4 ± 0.8	0.9 ± 0.6	0.056
Banff i score	1.0 ± 1.0	0.3 ± 0.7	0.005
Banff ci score	1.2 ± 0.7	2.1 ± 0.9	0.001
Banff ct score	1.2 ± 0.5	2.1 ± 0.9	0.001
Extent of SV40-T	1.8 ± 0.8	0.6 ± 0.5	<0.001

Abbreviations: ci, interstitial fibrosis; ct, tubular atrophy; i, interstitial inflammation; t, tubulitis.

#### Switching from tacrolimus to CsA postponed renal transplant function deterioration and graft loss

The mean serum creatinine level and eGFR of all 24 patients was relatively stable at each follow-up time-point (Figure 2). As compared with the time of diagnosis, serum creatinine, and eGFR were not significantly different at 12 and 24 months after switching to CsA (Table 2). The renal survival rate was 100% at last follow-up.

### Safety evaluation

#### Acute rejection events

The serum creatinine level of one patient increased to 300 µmol/l at 9 months after switching to CsA (viremia had become negative). Renal biopsy revealed ABMR combined with TCMR Banff IIa. After addition of methylprednisolone, and increasing the dose of both MMF and CsA, the serum creatinine level was 323 µmol/l at the 24-month follow-up. Another patient was confirmed as chronic active ABMR by the protocol biopsy at 12 months. The patient was treated with methylprednisolone, and the serum creatinine was 154 µmol/l at the 24-month follow-up. Rejection in both patients was due to non-adherence documented by missing medication, including cyclosporin, MPA, and prednisone. Neither patient developed a recurrence of viremia.

#### Other adverse events

Five patients developed proteinuria. Amongst them, four patients were confirmed as having recurrent IgA nephropathy through biopsy, and one patient was diagnosed with mixed rejection mentioned above. There was no CsA-related adverse event.

## Discussion

There are few controlled studies available to guide the management of BKVN in kidney transplant recipients [3]. Reducing immunosuppression is a relatively effective treatment. In a retrospective study, maintenance immunosuppression was reduced in 52 patients with biopsy-proven BKVN. Eight patients (15.3%) developed acute rejection, and eight patients (16.4%) lost their grafts [28]. Schaub et al. [29] treated 13 patients with definitive BKVN, 17 patients with presumptive BKVN, and 8 patients with low BKV-viremia by reducing the dosage of tacrolimus, and subsequently reducing the dose of MPA. Clearance of BKV-viremia was achieved in 35 of the 38 patients (92%), but 7 patients (18%) required treatment for presumed concurrent rejection while BKV-viremia was present. Only reducing immunosuppressive dosage might be insufficient or excessive. As compared with above studies, the therapeutic regimen in the present study was associated with a higher virus clearance rate, lower secondary rejection rate, and lower renal allograft loss rate.

Although both are calcineurin inhibitors, the immunosuppressive strength of CsA is weaker than that of tacrolimus [11]. Registry analyses, single-center retrospective studies, and prospective randomized clinical trials have suggested that tacrolimus is associated with a higher risk of BKV reactivation than CsA [14,15,17,18]. *In vitro* studies have indicated that tacrolimus activates BKV replication via FK-binding protein 12 in primary human tubular epithelial cells [19]. CsA inhibits BKV large T-antigen and viral protein 1 expression by binding to cyclophilins [17,18]. MPA has been demonstrated to be an important factor leading to BKV activation [14]. The combination of CsA with MPA results in less intense immunosuppression than that of tacrolimus with the same dose of MPA. This is due to CsA inhibition of the enterohepatic recirculation of the major MPA metabolite, glucuronide [30]. After switching from tacrolimus to low-dose CsA, the overall immunosuppressive intensity is reduced, and with the antiviral effect of CsA virus clearance is promoted.

In the present study, there was no statistical difference in the serum creatinine level at 24 months after switching to CsA and the time of diagnosis. The graft survival rate was 100%. The stability of renal transplant function reported in the present study is superior to that in previous studies [28,29]. The incidence of rejection events was 8.3%, which was lower than that reported in two other studies (15.3 and 18.0%, respectively) [28]. In this study, two patients developed rejection, which was correlated with drug incompliance.

Regardless of the treatment strategy employed for BKVN, rapid viremia clearance has been associated with stable or improving eGFR [31]. In this study, the viremia clearance time was 2.7 ± 2.0 months, which was shorter than that reported in other studies [20,29]. Of course, the shorter viremia clearance time in this study may be partially attributed to a threshold of 1000 copies/ml for viremia rather than a lower threshold, such as 200 copies/ml reported by Tong et al. [32]. It is worth noting that the clearance rate of decoy cells was the fastest. The massive replication of BKV in renal tubular epithelium and urinary tract epithelium could cause shedding of epithelial cells, which are shed into the urine to form decoy cells [33]. Negative decoy cells indicate that BKV replication is reduced or is completely blocked in the graft. In the present study, decoy cells became negative sooner than viremia became negative, which is consistent with the kinetics of BKV clearance.

From a pathological view, the evolution of BKVN may begin with BKV activation in transitional cells, followed by retrograde infection in the medullary collecting ducts, distal tubules, and proximal tubules [2]. Early BKV infection is mainly characterized by involvement of the medullary collecting ducts [2,4]. Medullary collecting duct function is closely correlated with the ability to concentrate urine [34]. Necrotic epithelia are shed into the collecting ducts after BKV infection, leading to impairment of the ability to concentrate urine, which is characterized by decreased urine specific gravity. Findings of the present study showed that urine specific gravity was significantly lower than normal level at the time BKVN was diagnosed. At 3 months after switching to CsA urine specific gravity had increased, and the trend of urine specific gravity increase was consistent with the trend of BKV load decrease in blood and urine. We speculate that after virus clearance in the graft, the epithelia of collecting duct repair, and function normally to concentrate urine. Urine specific gravity could be used to predict renal tubular injury and repair, as well as to predict the degree of virus clearance in BKVN patients.

In addition to decreasing viremia, diminishing or absent viral cytopathological changes and SV40-T staining usually indicates resolving BKVN [35]. In the present study, repeated biopsies showed that SV40-T staining became negative in 8 patients (42.1%), and was reduced in 11 patients (58.9%), suggesting resolving BKVN. Similar to other studies, repeated biopsies in the present study showed that residual tubulointerstitial inflammation can occur after virus clearance, and it is believed that this antiviral inflammatory microenvironment promotes healing and a good prognosis [35,36]. However, if tubulointerstitial inflammation is aggravated, which occurred in the non-positive area of SV40-T staining, TCMR should be taken into account [35,36]. In this study, repeated biopsies showed aggravated tubulitis and interstitial inflammation combined with *de novo* end arteritis in only one patient, who was thus diagnosed with TCMR Banff IIa mixed with ABMR.

There are some limitations of the present study. The sample size was small due to strict inclusion criteria. In addition, no control group that received a standardized treatment for BKVN was included.

The present study initially reveals that switching from tacrolimus to low-dose CsA could serve as an alternative treatment for early BKVN in kidney transplant recipients. This strategy still requires more large-scale, multicenter clinical studies to confirm.

## Clinical perspectives

- There is no effective therapy for BKVN. CsA has a lower immunosuppressive effect than tacrolimus. *In vitro* studies have shown that CsA inhibits BKV replication.
- We treated 24 renal transplant recipients with BKVN by switching from tacrolimus to low-dose CsA. The viremia in all patients cleared at a mean of 2.7 ± 2.0 months after switching to CsA. Renal biopsies at a mean of 11.2 months (range: 9.1–12.5 months) showed that 8 patients (42.1%) were negative for BKV, and 11 patients (58.9%) had a decrease in BKV load (*P*<0.001). There was no statistical differences in the serum creatinine level between the time of diagnosis and at 24 months (*P*=0.963). The graft survival rate was 100%. Only two patients (8.3%) had an acute rejection reaction.
- Switching from tacrolimus to low-dose CsA may serve as a safe and effective therapy for BKVN.

## Abbreviations

ABMR: antibody-mediated rejection
BKV: BK virus
BKVN: BKV nephropathy
CNI: Calcineurin inhibitor
CsA: cyclosporine A
eGFR: estimated glomerular filtration rate
MDRD: Modification of Diet in Renal Disease
MMF: mycophenolate mofetil
MPA: mycophenolic acid
TCMR: T-cell-mediated rejection
